# Doxycycline Induces Apoptosis and Inhibits Proliferation and Invasion of Human Cervical Carcinoma Stem Cells

**DOI:** 10.1371/journal.pone.0129138

**Published:** 2015-06-25

**Authors:** Binlie Yang, Yuping Lu, Ai Zhang, Aizhi Zhou, Lei Zhang, Lanrong Zhang, Limin Gao, Yuhua Zang, Xiuhua Tang, Liyan Sun

**Affiliations:** Department of Gynecology & Obstetrics, People Hospital of Pudong New Area, Shanghai, 20299, China; Rajiv Gandhi Centre for Biotechnology, INDIA

## Abstract

**Background:**

Cancer stem cells (CSCs) are proposed to be responsible for high recurrence rate in cervical carcinoma. Reagents that can suppress the proliferation and differentiation of CSCs would provide new opportunities to fight against tumor recurrence. Doxycycline has been reported as a potential anti-cancer compound. However, few studies investigated its inhibitory effect against cervical cancer stem cells.

**Methods:**

HeLa cells were cultured in cancer stem cell conditional media in a poly-hema-treated dish. In this non-adhesive culture system, HeLa cells were treated with cisplatin until some cells survived and formed spheroids, which were then collected and injected into the immunodeficient mice. Cisplatin was administered every three days for five times. The tumor xenografts with CSC enrichment were cultured in cancer stem cell specific medium again to form tumorsphere, which we called HeLa-CSCs. Expression of cancer stem cell markers in HeLa-CSCs was measured by flow cytometry and qPCR. HeLa-CSCs were then treated with doxycycline. Proliferation and differentiation rates were determined by the size of spheres formed *in vitro* and tumor formed *in vivo*.

**Results:**

We developed a new strategy to selectively enrich CSCs from human cervical carcinoma cell line HeLa, and these HeLa-CSCs are CD133+/CD49f+ cell populations with significantly enhanced expression of stem cell markers. When these HeLa-CSCs were treated with doxycycline, the colony formation, proliferation, migration and invasion, and differentiation were all suppressed. Meanwhile, stem cell markers SOX-2, OCT-4, NANOG, NOTCH and BMI-1 decreased in doxycycline treated cells, so as the surface markers CD133 and CD49f. Furthermore, proliferation markers Ki67 and PCNA were also decreased by doxycycline treatment in the *in vivo* xenograft mouse model.

**Conclusions:**

Cancer stem cells are enriched from sphere-forming and chemoresistant HeLa-derived tumor xenografts in immunodeficient mice. Doxycycline inhibits proliferation, invasion, and differentiation, and also induces apoptosis of these HeLa-CSCs *in vitro* and *in vivo*.

## Introduction

Carcinoma of the uterine cervix is the second most common malignancy that affects women worldwide and causes high mortality; approximately 500,000 new cases and more than 270,000 deaths occur each year due to this disease [1, 2]. Cervical cancer (CC) is more prevalent among women of low socioeconomic status and is a major health problem in developing countries. Cancer stem cells (CSCs), from which tumors often arise, are considered as an important target for developing future cancer therapies or improving the current therapies [3–5]. However, CSCs are multi-drug resistant and also resistant to other current therapies. Hence it is important to identify cervical cancer stem cells-related biomarkers and drug targets.

While it is well known that self-renewal and differentiation capacity are hallmark traits of normal stem cells (SCs), tumor cells are also found to possess the high proliferative capacity and phenotypic plasticity [3]. These similarities have given rise to the hypothesis of the cancer stem cells (CSCs), a subpopulation of cancer cells derived from normal SCs possessing tumor-initiating capability [6–8]. Importantly, it is not known whether the undifferentiated stage in tumor cells reflects the possession of SC-like traits. The isolation of putative CSCs has been achieved successfully via the use of various techniques. One key molecular characteristic of CSCs is their capability of extensive proliferation and clone formation in suspension culture [9], and tumor sphere-forming assays have been widely applied to identify potential CSC populations [10]. There were some reports suggesting CD133, CD44, EPCAM and CD90 as molecular markers of CSCs [11–14], and CSCs can also be isolated using flow cytometry according to the expression of these specific cell surface markers, such as CD133 and CD44 [15, 16]. However, it is still quite challenging and time-consuming to identify CSC cells by simply using these markers. Up to this date, therapeutic method targeting the cancer stem cell is not yet available.

As a second-generation derivative of tetracyclines, doxycycline (Doc) is commonly used to treat a variety of infections. Many reports have demonstrated that doxycycline is a pluripotent drug with anticarcinogenic functions by inhibiting cell growth and inducing apoptosis in tumor cells, including an anti-tumor growth effect on human oral squamous-cell carcinoma and the inhibition of migration of melanoma cells. Doxycycline also has a potential for enhancing therapeutic activity of biological cancer therapies [17]. Doc has been known for its matrix metalloproteinase inhibitor (MMPi) activity. However, the effect of Doc on cervical cancer stem cells remains unknown.

In this study, we developed a new strategy to enrich CSCs in human cervical carcinoma cells line, HeLa cells. Using these CSCs as a model, we tested the effect of Doc on the proliferation and differentiation of CSCs and demonstrated its inhibitory activity in both processes.

## Materials and Methods

### Cell culture

HeLa cells were originally obtained from American type culture collection (ATCC) and stored in our laboratory. Cells were cultured in DMEM medium supplemented with 10% FBS at 37°C in a humidified atmosphere containing 5% CO_2_. The medium was changed every day. Cells were harvested with 0.25% trypsin/EDTA and suspended at a concentration of 5×10^5^ cells/ml in TSC medium (DMEM supplemented with 10 ng/ml EGF, 10 ng/ml bFGF, 10 ng/ml Noggin, and 1000 U/ml LIF). Cells were then cultured in 3.5-cm dishes precoated with poly-HEMA for 14 days in order to form colonies. The colonies were treated with 0 or 2 μg/ml cisplatin for 72 h respectively.

### *In vivo* tumor growth analysis

NOD-SCID nude mice, 4 weeks of age, were purchased from Shanghai Laboratory Animal Company. Mice were housed under pathogen-free conditions. All of the procedures of animal studies were approved by the Animal Care and Use Committee of Shanghai Tongji University (Permit Number: 20120007) and conducted in strict accordance with institutional guidelines. All efforts were made to minimize the number of animals used and to reduce their suffering. The HeLa colonies consisting of ~5×10^5^ cells were subcutaneously injected into the left flank of anesthetized nude mice. Cisplatin was then administrated by intraperitoneal injection 24 h later at the dosage of 1, 2 and 6 mg/kg body weight respectively. The tumor size was monitored daily for 18 days, and mice were euthanized by CO_2_ inhalation for tumor sample collection. Bioluminescence imaging of tumor was performed on anesthetized nude mice using an IVIS imaging system per manufacture's instruction (Xenogen Corp., Alameda, CA).

### Tissue disaggregation and sphere culture assay

Xenografted tumors were dissected, washed with PBS, minced into small pieces with sterile scalpels and subjected to enzymatic dissociation with trypsin repeatedly. Tumor cells were then resuspended into TSM medium (serum-free neural stem cell medium supplemented with 20 ng/ml EGF, 20 ng/ml bFGF, 10 ng/ml Noggin and 1000 U/ml LIF). The cells suspended in the medium were harvested 60 h later and cultured to allow for the formation of tumor sphere.

### Quantitative real time PCR (qPCR)

Two weeks after the formation of tumor-spheres, total RNA of the spheres was isolated using TRIzol (Invitrogen) according to manufacturer’s protocol. The absorbance ratio at 260/280nm of all the samples was quantified using the Nano-Drop ND-1000 spectrophotometer (Thermo Fisher Scientific). qPCR was performed on the LightCycle480 system (Roche) using SYBR Green Supermix (Takara). qPCR reaction condition was 30 s at 94°C followed with 50 cycles of, 5 s at 94°C and 30 s at 60°C. β-actin was used as internal control, and the sequences of the primers are readily available upon request.

### Immunocytochemistry

Cell colonies were fixed in 10% formalin for 20 min at RT. Formalin-fixed samples were washed twice with PBS, followed by incubation with the blocking solution (0.2% Triton-100 and 5% goat serum in PBS) for 1 h. Subsequently, samples were incubated with primary antibody and secondary antibody for 1 h respectively.

### Western blotting assay

Whole cell lysates were prepared using pre-chilled RIPA (50 mM Tris/HCl pH7.4, 150 mM NaCl, 1 mM EDTA, 1% Nonidet P-40, 0.1% SDS, 0.5% deoxycholate). The samples were then centrifuged at 12000 g for 20min at 4°C and supernatants were collected for protein concentration determination. The total proteins was separated on 10% SDS-PAGE gel, and transferred onto a nitrocellulose membrane (Millipore). The membrane was incubated with the blocking solution, followed by incubation overnight with appropriate primary antibodies at 4°C and subsequently with secondary antibodies for 1 h at RT. The signals were visualized using LI-COR infrared imaging system according to the manufacturer’s guidelines.

### Flow Cytometry

Dissociated cells were incubated with primary antibodies for 20 min on ice, washed twice with HBSS containing 2% FBS, and resuspended into HBSS containing 2% FBS and corresponding secondary antibodies for a 20-min incubation. After washing, flow cytometry was carried out using a FACSAria flow cytometer (BD Immunocytometry Systems). The antibodies used were anti-CD133 and anti-CD49f, each at a dilution of 1:40.

### Cell Invasion and Migration Assays

The invasive potential of the cancer stem cells were assayed using Transwells (8-mm pore size, Corning Costar Corp) placed in 24-well plates. First, for the cell invasion assay, 0.1 ml Matrigel (50 mg/ ml, BD Biosciences) was added onto the plate surface and incubated for 3 hrs before the supernatant was removed. Second, 200 μl of trypsin digested cell suspension (10^4^ cells) was added to the upper layer of each insert that was coated with Matrigel. Third, 450 μl of RPMI1640 containing 10% fetal bovine serum was added into the lower compartment, and the cells were allowed to invade for 48 hours at 37°C with 5% CO_2_. After incubation, the cells were fixed with 95% absolute alcohol and stained with crystal violet. The cells that had invaded into the bottom surface of the filter were counted and imaged under an inverted microscope (Olympus Corp. Tokyo, Japan) over 6 random fields in each well. Each experiment was performed 3 times. The cell migration assay followed essentially the same procedure as the invasion assay but without the use of Matrigel.

### Statistical Analysis

Data collected from each (experimental and control) group were expressed as mean ± SEM. One-way analysis of variance and unpaired Student’s t-test were performed to analyze the differences between groups using GraphPad Prism 5 program and p<0.01 was denoted as statistically significant difference between experimental and control groups.

## Results

### Cisplatin enriched the HeLa derived CSCs

**Fig 1:**
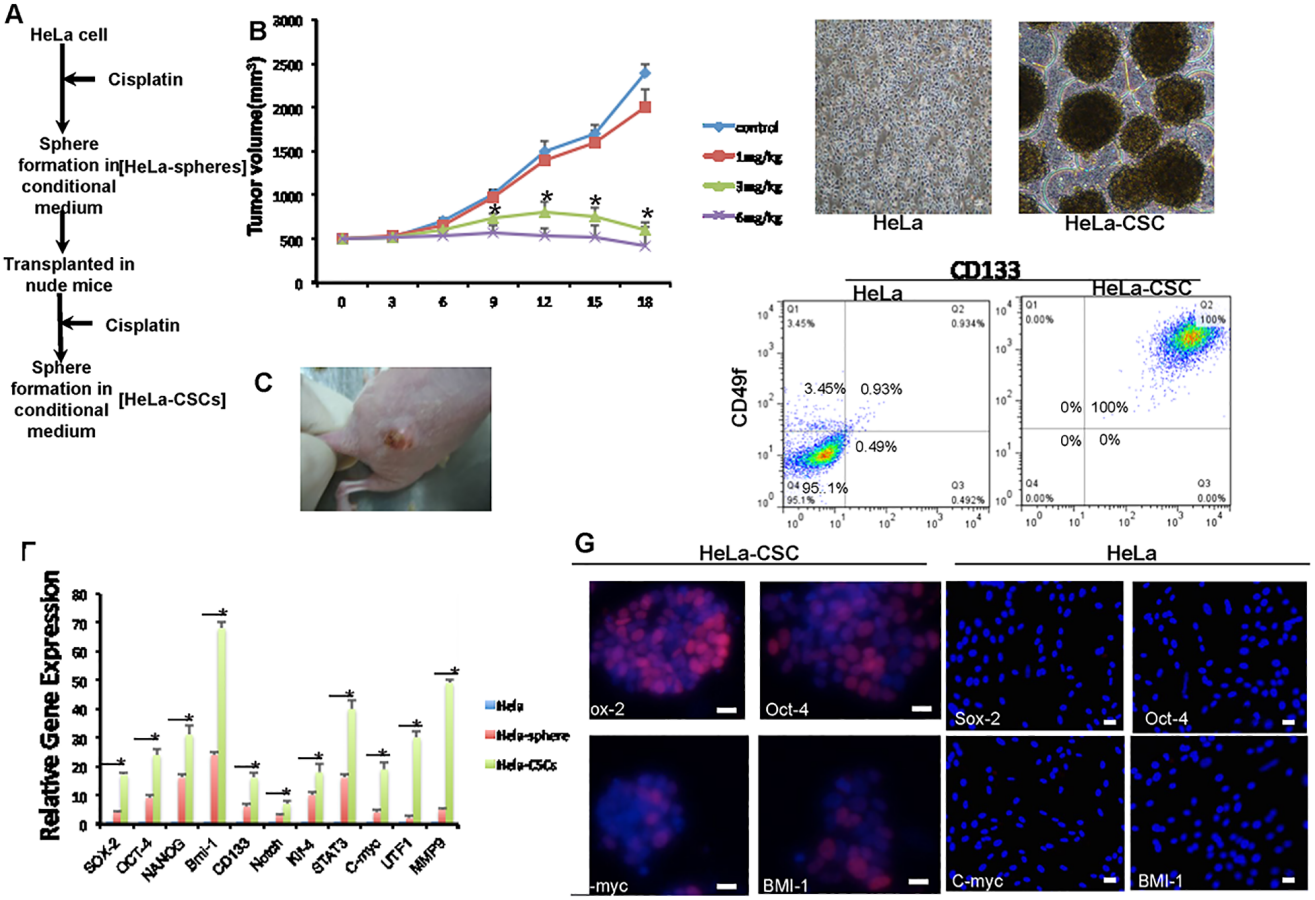
Cisplatin enriched CSCs derived from HeLa cells. **A:** The workflow of HeLa-CSC enrichment and isolation. B-C: Tumor development in response to various dose of cisplatin administrated every 3 days. The quantification of tumor volume (B) and representative image (C) were shown. n = 3 for each group, and * indicates p<0.01 compared to control group. D-E: The morphology (D) and expression of surface markers CD133 and CD49f according to flow cytometry (E) in isolated HeLa-CSCs. F-G: Expression levels of classic stem cell markers in HeLa-CSCs as measured by qPCR (F). n = 3 for each group, and * indicates p<0.01. Expression of 4 markers was confirmed by immunofluorescence staining (G).

The workflow of HeLa-CSCs enrichment and isolation was illustrated in Fig 1A. When grown in suspension culture system with stem cell specific media and polyhema-precoated dishes, some of the suspended HeLa cells aggregated and then formed spheres after 14 days. Such sphere formation indicated an enrichment of stem cell-like population. Spheres (~5×10^5^ cells) obtained were injected into nude mice and tumors were detected 2 weeks later. Subsequently, we treated the mice with different concentrations of cisplatin (1mg/kg, 3 mg/kg and 6 mg/kg) every 3 days. The size of xenografted tumors in mice was reduced by 6 mg/kg cisplatin treatment (Fig 1B). Xenografted tumors (Fig 1C) from mice treated with 6mg/kg cisplatin were dissociated into single cells and then cultured in the non-adhesive suspension culture system, or cultured in DMEM medium with 10% FBS (Fig 1D, left) and CSC conditional medium (Fig 1D, right). A portion of cells attached to the culture plates 48h later. Using flow cytometry, we found that these cells expressed much higher levels of the surface markers CD133 and CD49f (Fig 1E).

### HeLa-CSC sphere cells express stem cell markers

To further determine whether these spheres isolated from the cisplatin resistant tumors (HeLa-CSCs) have properties of stem cells, we carried out quantitative real-time PCR to measure the expression levels of several classic stem cell markers NANOG, OCT-4, SOX-2, NOTCH and CD133. Compared to untreated HeLa cells, the expression levels of stem cell markers were all up-regulated in HeLa-CSCs (Fig 1F and 1G).

### Doxycycline inhibits the clone formation and induces apoptosis of HeLa-CSCs

**Fig 2:**
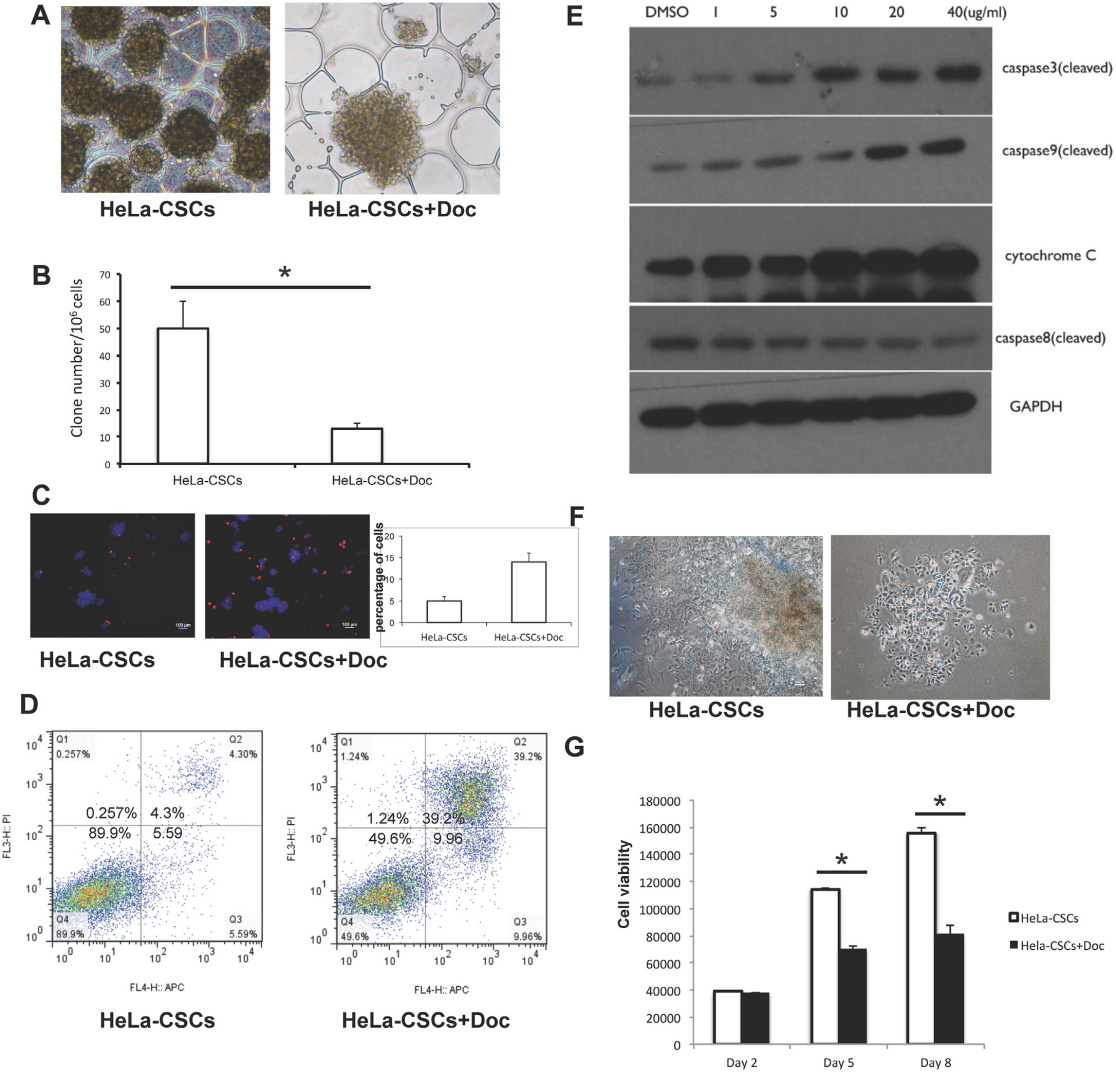
Doxycycline inhibits colony formation and induces apoptosis of HeLa-CSCs. A-B: 20 μg/ml doxycycline were added to Cisplatin enriched HeLa-CSCs, the morphology of HeLa-CSCs is shown (A) and colony number were measured (B). * indicates p<0.01. C-D: Apoptosis of doxycycline treated HeLa-CSCs were analyzed using TUNEL assay (C) and ANNEXIN V-PI staining (D). E: Expression of active caspase 3, caspase 8, caspase 9, and cytochrome C as determined by Western blotting. F: Representative images showing morphology of HeLa-CSCs before and after a 20 μg/mL Doc treatment for 3 days. G: Cell viability of differentiated HeLa-CSCs in DMEM+10%FBS medium with and without doxycycline at day 3. n = 3 for each group, and * indicates p<0.01.

When HeLa-CSCs were treated with 20 μg/ml doxycycline, some of the colonies were dispersed (Fig 2A), and clone number significantly decreased (Fig 2B). TUNEL staining showed higher rate of cell apoptosis in doxycycline treated clones (Fig 2C). This increase of apoptosis was further confirmed with ANNEXINV-PI assay (Fig 2D). To determine apoptosis-related caspase activity, active caspase 3, caspase 8, caspase 9, and cytochrome C were assayed by Western blotting, which showed that caspase 3 and caspase 9 were activated in doxycycline induced apoptosis (Fig 2E). Colony formation and viability of HeLa-CSCs were monitored after doxycycline treatment for 8 days, and both were significantly decreased during the period of culture (Fig 2F and 2G).

### Expression levels of stem cell markers were reduced in doxycycline treated HeLa-CSCs

**Fig 3:**
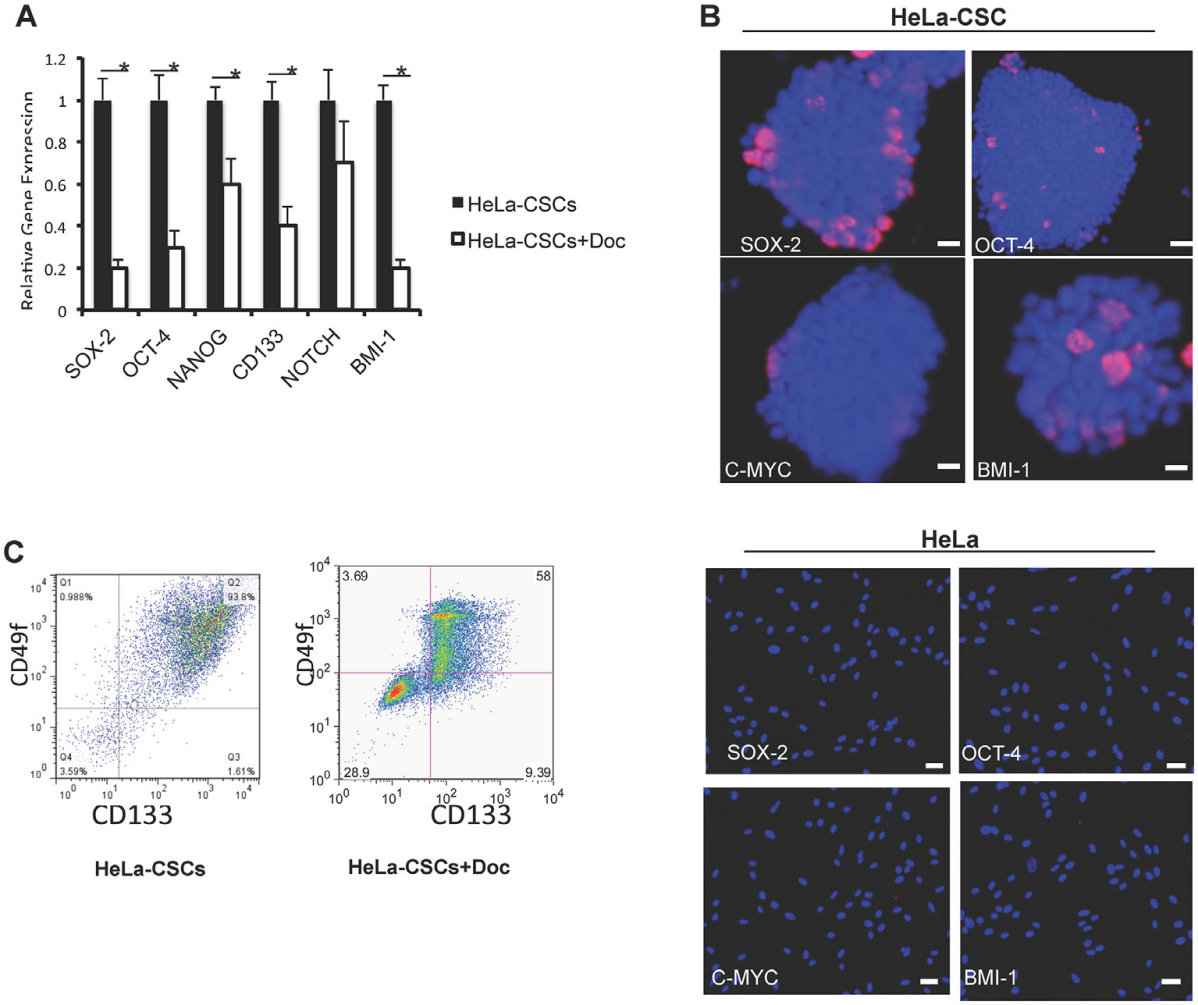
Expression levels of stem cell markers were reduced in doxycycline treated HeLa-CSCs. A: Expression levels of classic stem cell markers in doxycycline treated HeLa-CSCs measured by qPCR. n = 3 for each group, and * indicates p<0.01. B: Expression of stem cell markers were confirmed by immunofluorescence staining. C: Expression of surface markers of HeLa-CSCs and HeLa-CSCs+Doc was determined using flow cytometry.

To examine whether doxycycline affects the stem characteristic of HeLa-CSCs, we assayed for the expression of classic stem cell markers including SOX-2, OCT-4, NANOG, CD133, NOTCH and BMI-1 using qPCR, and the mRNA levels of all these markers were decreased in doxycycline treated HeLa-CSCs (Fig 3A), which were further confirmed by immunocytochemistry (Fig 3B). Consistent with the decreased expression of stem cell marker genes, surface markers CD133 and CD49f of HeLa-CSCs were also decreased as determined by flow cytometry assay (Fig 3C).

### EMT and migration were inhibited in doxycycline treated HeLa-CSCs

**Fig 4:**
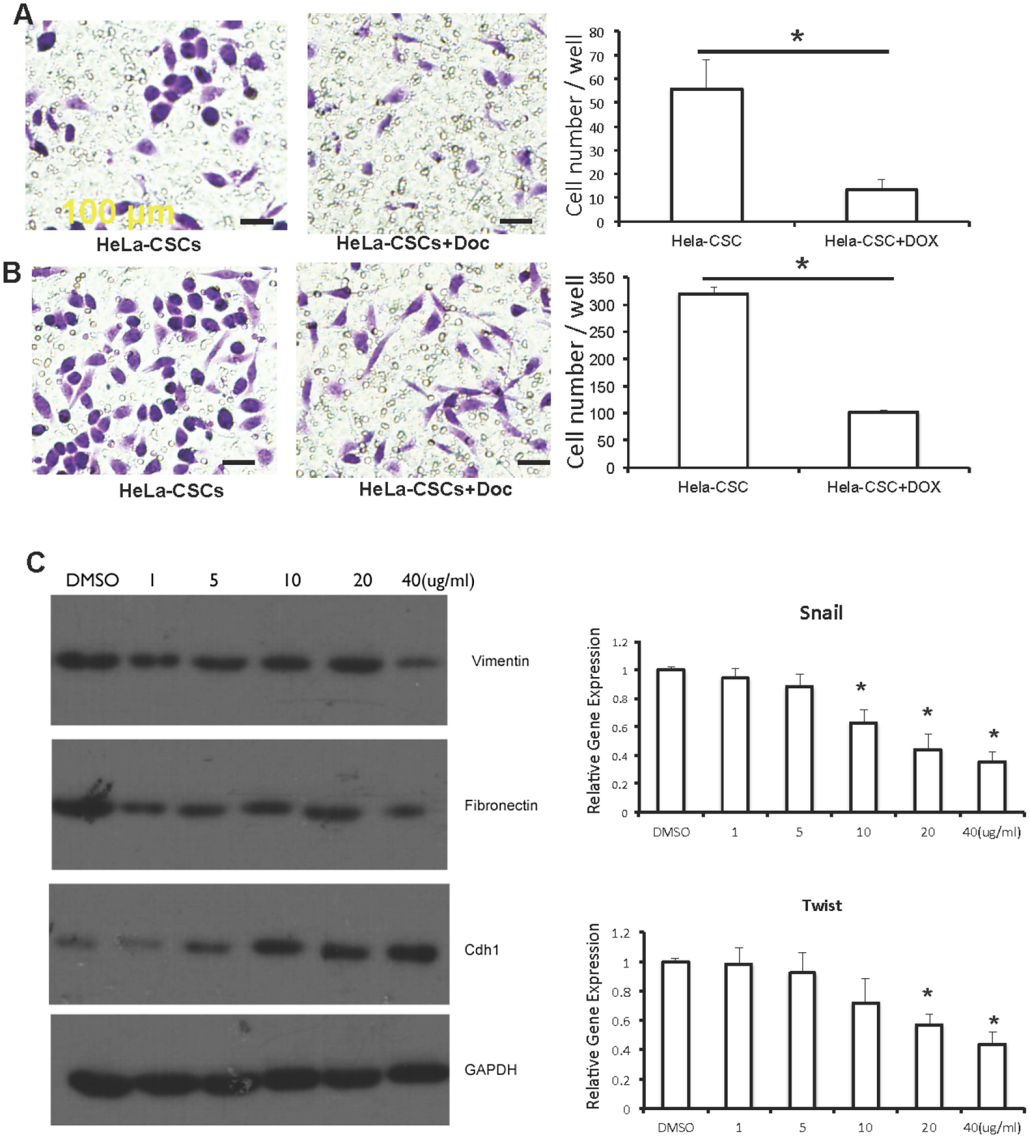
EMT was inhibited in Doc treated HeLa-CSCs. A-B: Transwell assay were applied to monitor the migration (A) and invasion (B) of Doc treated HeLa-CSCs. n = 3 for each group, and * indicates p<0.01. C: EMT markers were detected by Western blotting. D: The expression level of Snail and Twist were detected by Real-time qPCR. * indicates p<0.01.

Transwell assays were applied to monitor the migration and invasion of doxycycline treated HeLa-CSCs, and showed that both migration and invasion of HeLa-CSCs were inhibited when treated with doxycycline (Fig 4A and 4B). Furthermore, the expression of EMT markers was detected by Western blotting or qPCR. Decreased expression of Vimentin and Fibronectin in 40 μg/ml doxycycline while increased expression of CDH1 in as low as 10 μg/ml doxycycline were detected (Fig 4C), suggesting that doxycycline inhibited EMT of HeLa-CSCs. The expression of Snail and Twist were also inhibited by doxycycline treatment (Fig 4D).

### Doxycycline reduces the tumor growth capacity of HeLa-CSCs *in vivo*

**Fig 5:**
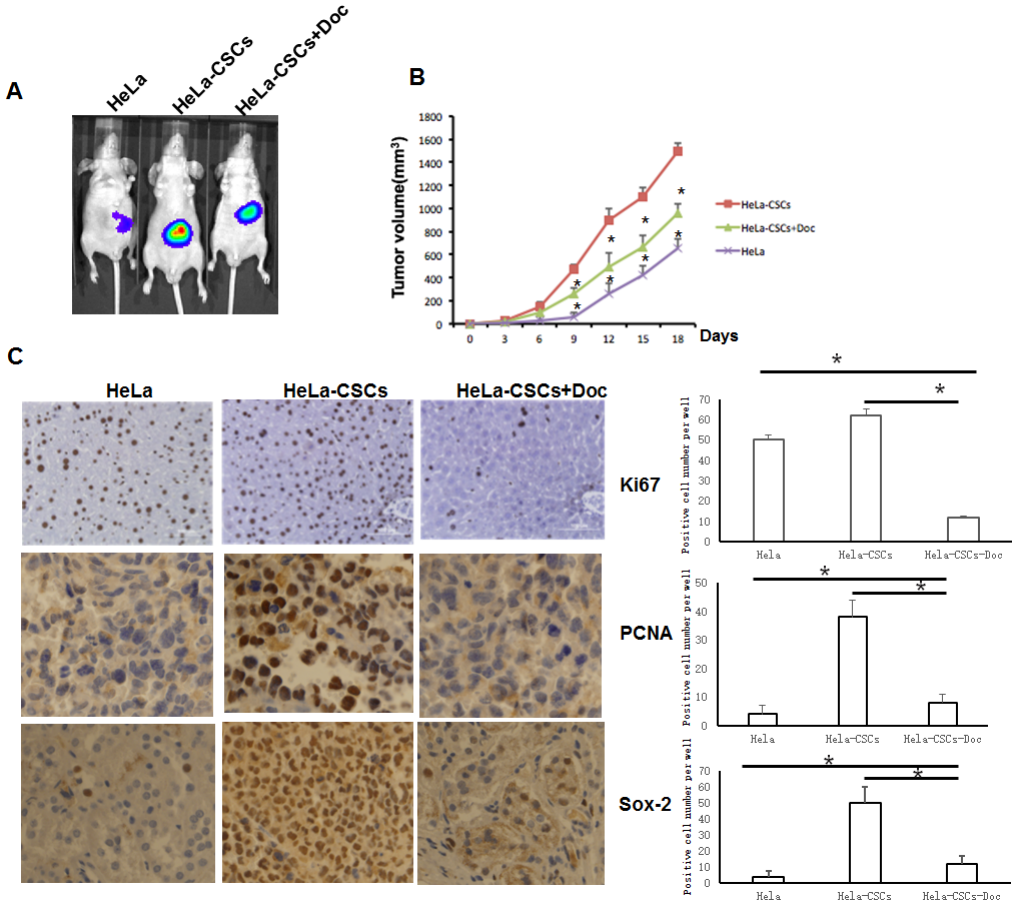
*In vivo* tumor formation of Doc treated HeLa-CSCs. A: IVIS imaging of tumor growth 18 days after the xenograft in NOD-SCID mice. B: The tumor volume during the culture. n = 3 for each group, and * indicates p<0.01 compared to HeLa-CSCs group. C: Cell proliferation was evaluated by PCNA, Ki67 and SOX-2 immunohistochemistry in xenografts. D: Statistical analysis of PCNA, Ki67 and SOX-2 positive cells from panel C, * indicates p<0.01.

To examine the tumor growth potential of HeLa-CSCs, 5 x10^5^ of HeLa cells, HeLa-CSCs, or 40 μg/ml Doc-pretreated HeLa-CSCs were injected into NOD-SCID mice and the tumor volume was measured. There were significant differences of tumor volume among the three groups (Fig 5A and 5B). Doxycycline pretreated HeLa-CSCs had drastically reduced capacity of tumor growth. Meanwhile, immunohistochemistry assays revealed that the stem cell maker SOX-2, proliferation marker PCNA and Ki67 all markedly decreased in doxycycline pre-treated xenografts (Fig 5C and 5D).

## Discussion

In this study, we successfully established conditions to isolate and sustain CSC populations derived from HeLa cells. CSC populations are capable of forming tumor. Doxycycline can inhibit proliferation and differentiation rate of the isolated HeLa-CSCs (Figs 2 and 4), and prevent tumor formation, thus potentially enabling a more effective chemotherapy (Fig 5).

Drug resistance is still the major cause of failure in chemotherapy. The drug-resistant tumors eventually induce relapse in a large proportion of cases. The high rate of tumor relapse may be due to the inability of drugs to kill a small subpopulation of cells (likely the CSCs), which can be resistant to the drugs [18]. Indeed, as few as one hundred cells expressing certain molecular markers can successfully establish tumors when transplanted into the NOD-SCID mice, while tens of thousands of cancer cells that express a different marker set failed to form tumors [19]. These studies suggest that only a small subset of cells in tumor is capable of tumor formation in such transplantation assays, and that CSCs may be the major origin of tumor. Consequently, the development of reliable models of CSCs is crucial for both basic and clinical cancer research.

Over the past 30 years, accumulating studies of cervical cancer have greatly widened our knowledge about this aggressive malignancy. However, the survival rate of patients fails to improve and has reached a plateau phase [20]. This is at least partly owing to our poor understanding of the complex molecular mechanisms underlying cervical cancer development and progression. The emerging CSC theory has shed more light on the study of cervical cancer, which would certainly benefit from the establishment of a reliable and representative CSC model of the disease.

There are commonly three methods for isolation of CSCs: a) cell sorting based on the expression of specific cell surface markers [21–23], b) sorting a side population (SP) based on Hoechst dye efflux [24], and c) sphere-formation culture [25]. The most common strategy is to start with surface marker-based cell sorting, followed by evaluation of self-renewal capability both *in vitro* and *in vivo*. The entire process of CSC identification and isolation is technically challenging and very time-consuming. Previous studies have suggested that CSCs could be isolated based on their drug resistance property from tumors such as breast and lung cancers [17, 22]. Here, we developed a new strategy through combining these methods together. Firstly, we generated tumor spheres by suspension culture, which was then injected into nude mice. Increasing resistance to cisplatin as well as the severity of the adverse effects limit the use of this drug for cervical cancer treatment, in order to know how to maintain its cancer-specific cytotoxic action, we suspect cisplatin can enrich the cancer stem cells, and we found cisplatin can enhance the sphere formation in this study, and these cells exhibit chemoresistance and express high levels of stem cell-related transcription factors. We further detected the expression patterns of several cell surface markers and confirmed the stem cell-like properties of the isolated sphere-formation cells.

Doxycycline has been used in clinical trials and many studies have investigated the underlying molecular mechanisms of anti-tumor effect of Doxycycline [26–30]. Combination of Doxycycline with other anti-cancer drugs was also shown to enhance the antitumor efficacy in several tumor models [31–33]. The effect of doxycycline on CSCs has not been reported before. Our study shows that doxycycline can inhibit the proliferation and differentiation rate of HeLa cell derived CSCs. This inhibitory effect makes doxycycline a good candidate as a chemosensitization component of the cocktail therapy in combination with other anticancer drugs to improve the efficacy of anti-cancer chemotherapy. However, the detailed mechanisms underlying doxycycline’s growth inhibition effect on HeLa-CSCs need further investigation.

In conclusion, in the present study, we established a new and efficient approach for isolating CSCs from HeLa cells. Moreover, we determined doxycycline as a potent agent in inhibiting the proliferation and differentiation of HeLa-CSCs. Our data may shed light upon the potential clinical utility of this compound in treating cervical cancer.
