# Correction: Doxycycline Induces Apoptosis and Inhibits Proliferation and Invasion of Human Cervical Carcinoma Stem Cells

**DOI:** 10.1371/journal.pone.0134201

**Published:** 2015-07-24

**Authors:** Binlie Yang, Yuping Lu, Ai Zhang, Aizhi Zhou, Lei Zhang, Lanrong Zhang, Limin Gao, Yuhua Zang, Xiuhua Tang, Liyan Sun

There is an omission in the Funding section. The correct funding information is as follows: This research was supported by the Pudong New District Health Systems Personnel Training Plan (NO. PWR1210–04) and Outstanding Leaders Training Program of Pudong Health Bureau of Shanghai (Grant NO. PWR1210-04). The funders had no role in study design, data collection and analysis, decision to publish, or preparation of the manuscript.
